# Supplementary material for: Transcriptome Analysis of the Immortal Human Keratinocyte HaCaT Cell Line Damaged by Tritiated Water
Source: Biology (Basel). 2023 Mar 3;12(3):405. doi: 10.3390/biology12030405 (PMC10045445; doi:10.3390/biology12030405)
Supplement: Supplementary file 1 [file biology-12-00405-s001.zip › biology-2135777-supplementary.pdf]

Supplementary Information

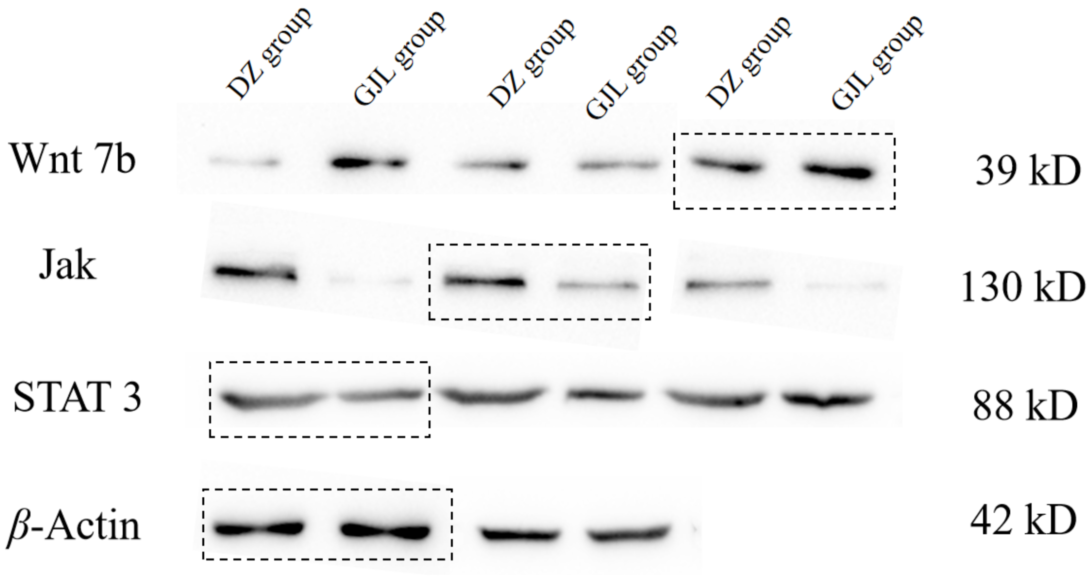

SI Figure S1. Full unedited western blot for Figure. 6 (A).

SI Table S1. DEGs on the sides and top in for Figure. 3 (A)

Gene ID	Gene Symbol	log_2 FC	$-log_{10}$ (Q value)	category
107987457	LOC107987457	-8.09	27.20	Down
100526740	ATP 5MF-PT CD1	-7.36	17.41	Down
100526832	PHOSPHO2-KLHL23	7.09	15.09	Up
54921	CHTF8	6.65	11.37	Up
56100	PCDHGB6	5.29	8.98	Up
6818	SULT1A3	1.77	300.00	Up

SI Table S2. Go ecrichment of list

Part	ID	Description	GeneRatio	BgRatio	pvalue	qvalue	geneID
BP	GO:0009812	flavonoid metabolic process	2/10	15/18862	2.65E-05	3.71E-03	445329/54576
BP	GO:0006805	xenobiotic metabolic process	2/10	120/18862	1.75E-03	4.15E-02	445329/54576
BP	GO:0071466	cellular response to xenobiotic stimulus	2/10	125/18862	1.89E-03	4.15E-02	445329/54576
BP	GO:0009410	response to xenobiotic stimulus	2/10	131/18862	2.08E-03	4.15E-02	445329/54576
BP	GO:1901215	negative regulation of neuron death	2/10	195/18862	4.53E-03	4.15E-02	7015/445329
BP	GO:0019614	catechol-containing compound catabolic process	1/10	10/18862	5.29E-03	4.15E-02	445329
BP	GO:0042424	catecholamine catabolic process	1/10	10/18862	5.29E-03	4.15E-02	445329

BP	GO:0030422	production of siRNA involved in RNA interference	1/10	11/18862	5.82E-03	4.15E-02	7015
BP	GO:0052697	xenobiotic glucuronidation	1/10	11/18862	5.82E-03	4.15E-02	54576
BP	GO:0006068	ethanol catabolic process	1/10	13/18862	6.87E-03	4.15E-02	445329
BP	GO:0019336	phenol-containing compound catabolic process	1/10	13/18862	6.87E-03	4.15E-02	445329
BP	GO:1902570	protein localization to nucleolus	1/10	14/18862	7.40E-03	4.15E-02	7015
BP	GO:1903799	negative regulation of production of miRNAs involved in gene silencing by miRNA	1/10	15/18862	7.93E-03	4.15E-02	7015
BP	GO:0034310	primary alcohol catabolic process	1/10	16/18862	8.45E-03	4.15E-02	445329
BP	GO:0034504	protein localization to nucleus	2/10	275/18862	8.82E-03	4.15E-02	7015/729540
BP	GO:0016246	RNA interference	1/10	17/18862	8.98E-03	4.15E-02	7015
BP	GO:0090399	replicative senescence	1/10	17/18862	8.98E-03	4.15E-02	7015
BP	GO:0070200	establishment of protein localization to telomere	1/10	18/18862	9.50E-03	4.15E-02	7015
BP	GO:0031647	regulation of protein stability	2/10	295/18862	1.01E-02	4.15E-02	7015/8293
BP	GO:0006607	NLS-bearing protein import into nucleus	1/10	20/18862	1.06E-02	4.15E-02	729540
BP	GO:0052695	cellular glucuronidation	1/10	20/18862	1.06E-02	4.15E-02	54576
BP	GO:1901214	regulation of neuron death	2/10	302/18862	1.06E-02	4.15E-02	7015/445329
BP	GO:0060965	negative regulation of gene silencing by miRNA	1/10	21/18862	1.11E-02	4.15E-02	7015
BP	GO:0006067	ethanol metabolic process	1/10	22/18862	1.16E-02	4.15E-02	445329
BP	GO:2000648	positive regulation of stem cell proliferation	1/10	22/18862	1.16E-02	4.15E-02	7015
BP	GO:2000773	negative regulation of cellular senescence	1/10	22/18862	1.16E-02	4.15E-02	7015
BP	GO:0005980	glycogen catabolic process	1/10	23/18862	1.21E-02	4.15E-02	8987
BP	GO:0060149	negative regulation of posttranscriptional gene silencing	1/10	23/18862	1.21E-02	4.15E-02	7015
BP	GO:0060967	negative regulation of gene silencing by RNA	1/10	23/18862	1.21E-02	4.15E-02	7015
BP	GO:1904754	positive regulation of vascular associated smooth muscle cell migration	1/10	23/18862	1.21E-02	4.15E-02	7015
BP	GO:0008202	steroid metabolic process	2/10	329/18862	1.24E-02	4.15E-02	445329/54576
BP	GO:0009251	glucan catabolic process	1/10	24/18862	1.27E-02	4.15E-02	8987
BP	GO:0051000	positive regulation of nitric-oxide synthase activity	1/10	24/18862	1.27E-02	4.15E-02	7015
BP	GO:0051923	sulfation	1/10	24/18862	1.27E-02	4.15E-02	445329
BP	GO:0006063	uronic acid metabolic process	1/10	25/18862	1.32E-02	4.15E-02	54576
BP	GO:0019585	glucuronate metabolic process	1/10	25/18862	1.32E-02	4.15E-02	54576
BP	GO:0034035	purine ribonucleoside bisphosphate	1/10	25/18862	1.32E-02	4.15E-02	445329

		metabolic process					
BP	GO:0044247	cellular polysaccharide catabolic process	1/10	25/18862	1.32E-02	4.15E-02	8987
BP	GO:0050427	3'-phosphoadenosine 5'-phosphosulfate metabolic process	1/10	25/18862	1.32E-02	4.15E-02	445329
BP	GO:1903798	regulation of production of miRNAs involved in gene silencing by miRNA	1/10	25/18862	1.32E-02	4.15E-02	7015
BP	GO:1990000	amyloid fibril formation	1/10	25/18862	1.32E-02	4.15E-02	8293
BP	GO:0070997	neuron death	2/10	342/18862	1.34E-02	4.15E-02	7015/445329
BP	GO:0022616	DNA strand elongation	1/10	26/18862	1.37E-02	4.15E-02	7015
BP	GO:0042634	regulation of hair cycle	1/10	26/18862	1.37E-02	4.15E-02	7015
BP	GO:0070920	regulation of production of small RNA involved in gene silencing by RNA	1/10	26/18862	1.37E-02	4.15E-02	7015
BP	GO:0000272	polysaccharide catabolic process	1/10	27/18862	1.42E-02	4.15E-02	8987
BP	GO:0035235	ionotropic glutamate receptor signaling pathway	1/10	27/18862	1.42E-02	4.15E-02	445329
BP	GO:0070199	establishment of protein localization to chromosome	1/10	27/18862	1.42E-02	4.15E-02	7015
BP	GO:0090344	negative regulation of cell aging	1/10	28/18862	1.47E-02	4.17E-02	7015
BP	GO:0045939	negative regulation of steroid metabolic process	1/10	29/18862	1.53E-02	4.17E-02	54576
BP	GO:0042402	cellular biogenic amine catabolic process	1/10	30/18862	1.58E-02	4.17E-02	445329
BP	GO:0070198	protein localization to chromosome, telomeric region	1/10	30/18862	1.58E-02	4.17E-02	7015
BP	GO:1904837	beta-catenin-TCF complex assembly	1/10	31/18862	1.63E-02	4.17E-02	7015
BP	GO:0009310	amine catabolic process	1/10	32/18862	1.68E-02	4.17E-02	445329
BP	GO:1901099	negative regulation of signal transduction in absence of ligand	1/10	32/18862	1.68E-02	4.17E-02	7015
BP	GO:2001240	negative regulation of extrinsic apoptotic signaling pathway in absence of ligand	1/10	32/18862	1.68E-02	4.17E-02	7015
BP	GO:0032770	positive regulation of monooxygenase activity	1/10	33/18862	1.74E-02	4.17E-02	7015
BP	GO:0042573	retinoic acid metabolic process	1/10	33/18862	1.74E-02	4.17E-02	54576
BP	GO:0045922	negative regulation of fatty acid metabolic process	1/10	34/18862	1.79E-02	4.17E-02	54576
BP	GO:2000352	negative regulation of endothelial cell apoptotic process	1/10	34/18862	1.79E-02	4.17E-02	7015
BP	GO:0060969	negative regulation of gene silencing	1/10	37/18862	1.94E-02	4.44E-02	7015
BP	GO:0042417	dopamine metabolic process	1/10	38/18862	2.00E-02	4.44E-02	445329
BP	GO:0046326	positive regulation of glucose import	1/10	38/18862	2.00E-02	4.44E-02	7015
BP	GO:0007212	dopamine receptor signaling pathway	1/10	40/18862	2.10E-02	4.51E-02	445329
BP	GO:1902895	positive regulation of pri-miRNA	1/10	40/18862	2.10E-02	4.51E-02	7015

		transcription by RNA polymerase II					
BP	GO:0072091	regulation of stem cell proliferation	1/10	41/18862	2.15E-02	4.51E-02	7015
BP	GO:2001239	regulation of extrinsic apoptotic signaling pathway in absence of ligand	1/10	42/18862	2.21E-02	4.51E-02	7015
BP	GO:1904738	vascular associated smooth muscle cell migration	1/10	43/18862	2.26E-02	4.51E-02	7015
BP	GO:1904752	regulation of vascular associated smooth muscle cell migration	1/10	43/18862	2.26E-02	4.51E-02	7015
BP	GO:0010828	positive regulation of glucose transmembrane transport	1/10	45/18862	2.36E-02	4.51E-02	7015
BP	GO:0014911	positive regulation of smooth muscle cell migration	1/10	45/18862	2.36E-02	4.51E-02	7015
BP	GO:1900087	positive regulation of G1/S transition of mitotic cell cycle	1/10	45/18862	2.36E-02	4.51E-02	7015
BP	GO:0031648	protein destabilization	1/10	46/18862	2.41E-02	4.51E-02	8293
BP	GO:0097720	calcineurin-mediated signaling	1/10	46/18862	2.41E-02	4.51E-02	445329
BP	GO:0044275	cellular carbohydrate catabolic process	1/10	47/18862	2.46E-02	4.51E-02	8987
BP	GO:1904707	positive regulation of vascular associated smooth muscle cell proliferation	1/10	47/18862	2.46E-02	4.51E-02	7015
BP	GO:1904036	negative regulation of epithelial cell apoptotic process	1/10	50/18862	2.62E-02	4.51E-02	7015
BP	GO:1902893	regulation of pri-miRNA transcription by RNA polymerase II	1/10	51/18862	2.67E-02	4.51E-02	7015
BP	GO:0006584	catecholamine metabolic process	1/10	52/18862	2.72E-02	4.51E-02	445329
BP	GO:0009712	catechol-containing compound metabolic process	1/10	52/18862	2.72E-02	4.51E-02	445329
BP	GO:0035196	production of miRNAs involved in gene silencing by miRNA	1/10	52/18862	2.72E-02	4.51E-02	7015
BP	GO:0050999	regulation of nitric-oxide synthase activity	1/10	52/18862	2.72E-02	4.51E-02	7015
BP	GO:0061614	pri-miRNA transcription by RNA polymerase II	1/10	52/18862	2.72E-02	4.51E-02	7015
BP	GO:2000772	regulation of cellular senescence	1/10	52/18862	2.72E-02	4.51E-02	7015
BP	GO:0007215	glutamate receptor signaling pathway	1/10	53/18862	2.78E-02	4.51E-02	445329
BP	GO:0046164	alcohol catabolic process	1/10	55/18862	2.88E-02	4.51E-02	445329
BP	GO:0019748	secondary metabolic process	1/10	56/18862	2.93E-02	4.51E-02	54576
BP	GO:0031050	dsRNA processing	1/10	56/18862	2.93E-02	4.51E-02	7015
BP	GO:0051353	positive regulation of oxidoreductase activity	1/10	56/18862	2.93E-02	4.51E-02	7015
BP	GO:0070918	production of small RNA involved in gene silencing by RNA	1/10	56/18862	2.93E-02	4.51E-02	7015
BP	GO:1902808	positive regulation of cell cycle G1/S phase transition	1/10	56/18862	2.93E-02	4.51E-02	7015

BP	GO:0002763	positive regulation of myeloid leukocyte differentiation	1/10	58/18862	3.03E-02	4.57E-02	9398
BP	GO:2000351	regulation of endothelial cell apoptotic process	1/10	58/18862	3.03E-02	4.57E-02	7015
BP	GO:0046324	regulation of glucose import	1/10	59/18862	3.09E-02	4.59E-02	7015
BP	GO:0090342	regulation of cell aging	1/10	60/18862	3.14E-02	4.60E-02	7015
BP	GO:0046686	response to cadmium ion	1/10	61/18862	3.19E-02	4.60E-02	7015
BP	GO:1903961	positive regulation of anion transmembrane transport	1/10	61/18862	3.19E-02	4.60E-02	7015
BP	GO:0061912	selective autophagy	1/10	62/18862	3.24E-02	4.63E-02	8987
BP	GO:0072089	stem cell proliferation	1/10	64/18862	3.34E-02	4.68E-02	7015
BP	GO:0072577	endothelial cell apoptotic process	1/10	64/18862	3.34E-02	4.68E-02	7015
BP	GO:0032768	regulation of monooxygenase activity	1/10	65/18862	3.39E-02	4.70E-02	7015
BP	GO:0036498	IRE1-mediated unfolded protein response	1/10	67/18862	3.50E-02	4.71E-02	445329
BP	GO:0038034	signal transduction in absence of ligand	1/10	67/18862	3.50E-02	4.71E-02	7015
BP	GO:0097192	extrinsic apoptotic signaling pathway in absence of ligand	1/10	67/18862	3.50E-02	4.71E-02	7015
BP	GO:0007004	telomere maintenance via telomerase	1/10	68/18862	3.55E-02	4.73E-02	7015
BP	GO:0042440	pigment metabolic process	1/10	72/18862	3.75E-02	4.93E-02	54576
BP	GO:0005977	glycogen metabolic process	1/10	74/18862	3.86E-02	4.93E-02	8987
BP	GO:0046323	glucose import	1/10	74/18862	3.86E-02	4.93E-02	7015
BP	GO:0006073	cellular glucan metabolic process	1/10	75/18862	3.91E-02	4.93E-02	8987
BP	GO:0006278	RNA-dependent DNA biosynthetic process	1/10	75/18862	3.91E-02	4.93E-02	7015
BP	GO:0044042	glucan metabolic process	1/10	75/18862	3.91E-02	4.93E-02	8987
BP	GO:0010827	regulation of glucose transmembrane transport	1/10	77/18862	4.01E-02	4.97E-02	7015
BP	GO:1901616	organic hydroxy compound catabolic process	1/10	77/18862	4.01E-02	4.97E-02	445329
BP	GO:0090398	cellular senescence	1/10	79/18862	4.11E-02	5.05E-02	7015
BP	GO:0010833	telomere maintenance via telomere lengthening	1/10	80/18862	4.16E-02	5.07E-02	7015
BP	GO:0014910	regulation of smooth muscle cell migration	1/10	81/18862	4.21E-02	5.08E-02	7015
BP	GO:0032092	positive regulation of protein binding	1/10	83/18862	4.32E-02	5.08E-02	7015
BP	GO:1904705	regulation of vascular associated smooth muscle cell proliferation	1/10	83/18862	4.32E-02	5.08E-02	7015
BP	GO:1990874	vascular associated smooth muscle cell proliferation	1/10	83/18862	4.32E-02	5.08E-02	7015
BP	GO:1900182	positive regulation of protein localization to nucleus	1/10	85/18862	4.42E-02	5.15E-02	7015
BP	GO:0006112	energy reserve metabolic process	1/10	87/18862	4.52E-02	5.23E-02	8987
BP	GO:0014909	smooth muscle cell migration	1/10	88/18862	4.57E-02	5.24E-02	7015

BP	GO:1903351	cellular response to dopamine	1/10	89/18862	4.62E-02	5.25E-02	445329
BP	GO:1903350	response to dopamine	1/10	90/18862	4.67E-02	5.25E-02	445329
BP	GO:0034502	protein localization to chromosome	1/10	91/18862	4.72E-02	5.25E-02	7015
BP	GO:1901992	positive regulation of mitotic cell cycle phase transition	1/10	91/18862	4.72E-02	5.25E-02	7015
BP	GO:0034308	primary alcohol metabolic process	1/10	94/18862	4.87E-02	5.33E-02	445329
BP	GO:1904035	regulation of epithelial cell apoptotic process	1/10	94/18862	4.87E-02	5.33E-02	7015
BP	GO:0048661	positive regulation of smooth muscle cell proliferation	1/10	96/18862	4.98E-02	5.35E-02	7015
CC	GO:0000782	telomere cap complex	1/11	13/19520	7.30E-03	2.88E-02	7015
CC	GO:0000783	nuclear telomere cap complex	1/11	13/19520	7.30E-03	2.88E-02	7015
CC	GO:0140445	chromosome, telomeric repeat region	1/11	13/19520	7.30E-03	2.88E-02	7015
CC	GO:0034045	phagophore assembly site membrane	1/11	15/19520	8.42E-03	2.88E-02	8987
CC	GO:0005697	telomerase holoenzyme complex	1/11	22/19520	1.23E-02	3.37E-02	7015
CC	GO:0000407	phagophore assembly site	1/11	31/19520	1.73E-02	3.95E-02	8987
CC	GO:0009295	nucleoid	1/11	44/19520	2.45E-02	4.19E-02	7015
CC	GO:0042645	mitochondrial nucleoid	1/11	44/19520	2.45E-02	4.19E-02	7015
CC	GO:0030315	T-tubule	1/11	51/19520	2.84E-02	4.31E-02	8987
CC	GO:0101003	ficolin-1-rich granule membrane	1/11	61/19520	3.39E-02	4.63E-02	8987
CC	GO:0070821	tertiary granule membrane	1/11	73/19520	4.04E-02	5.02E-02	8987
CC	GO:0005643	nuclear pore	1/11	85/19520	4.69E-02	5.35E-02	729540
MF	GO:0003964	RNA-directed DNA polymerase activity	1/11	12/18337	7.18E-03	4.54E-02	7015
MF	GO:0001972	retinoic acid binding	1/11	20/18337	1.19E-02	4.54E-02	54576
MF	GO:0070034	telomerase RNA binding	1/11	22/18337	1.31E-02	4.54E-02	7015
MF	GO:0030247	polysaccharide binding	1/11	25/18337	1.49E-02	4.54E-02	8987
MF	GO:0001223	transcription coactivator binding	1/11	26/18337	1.55E-02	4.54E-02	7015
MF	GO:0015020	glucuronosyltransferase activity	1/11	34/18337	2.02E-02	4.54E-02	54576
MF	GO:0034061	DNA polymerase activity	1/11	36/18337	2.14E-02	4.54E-02	7015
MF	GO:0042162	telomeric DNA binding	1/11	36/18337	2.14E-02	4.54E-02	7015
MF	GO:0005501	retinoid binding	1/11	37/18337	2.20E-02	4.54E-02	54576
MF	GO:0005504	fatty acid binding	1/11	37/18337	2.20E-02	4.54E-02	54576
MF	GO:0019840	isoprenoid binding	1/11	38/18337	2.26E-02	4.54E-02	54576
MF	GO:0034062	5'-3' RNA polymerase activity	1/11	43/18337	2.55E-02	4.54E-02	7015
MF	GO:0097747	RNA polymerase activity	1/11	43/18337	2.55E-02	4.54E-02	7015
MF	GO:0008146	sulfotransferase activity	1/11	52/18337	3.08E-02	5.09E-02	445329
MF	GO:0001221	transcription coregulator binding	1/11	61/18337	3.60E-02	5.38E-02	7015
MF	GO:0000049	tRNA binding	1/11	67/18337	3.95E-02	5.38E-02	7015
MF	GO:0016782	transferase activity, transferring sulfur-containing groups	1/11	69/18337	4.06E-02	5.38E-02	445329
MF	GO:0033293	monocarboxylic acid binding	1/11	71/18337	4.18E-02	5.38E-02	54576
